# Probable Transmission of Coxsackie B3 Virus from Human to Chimpanzee, Denmark

**DOI:** 10.3201/eid1807.111689

**Published:** 2012-07

**Authors:** Sandra C. Abel Nielsen, Tobias Mourier, Ulrik Baandrup, Tine Mangart Søland, Mads Frost Bertelsen, M. Thomas P. Gilbert, Lars Peter Nielsen

**Affiliations:** University of Copenhagen, Copenhagen, Denmark (S.C.A. Nielsen, T. Mourier, M.T.P. Gilbert, L.P. Nielsen);; Aalborg University, Aalborg, Denmark (U. Baandrup, L.P. Nielsen);; Copenhagen Zoo, Copenhagen (T.M. Søland, M.F. Bertelsen);; and Statens Serum Institut, Copenhagen (L.P. Nielsen)

**Keywords:** viruses, coxsackie B3 virus, coxsackievirus, nonhuman primate, human, chimpanzee, myocarditis, Copenhagen Zoo, Denmark, transmission, zoonoses

## Abstract

In 2010, a chimpanzee died at Copenhagen Zoo following an outbreak of respiratory disease among chimpanzees in the zoo. Identification of coxsackie B3 virus, a common human pathogen, as the causative agent, and its severe manifestation, raise questions about pathogenicity and transmissibility among humans and other primates.

Six serotypes of coxsackie B viruses (CBVs) (family *Picornaviridae,* genus *Enterovirus*) are recognized: CB1–6. CBV infections are common in humans and usually cause minor symptoms. However, CBVs are also linked to several serious acute manifestations in infants, children, and adults. CBVs are one of the most common causes of meningitis and can also cause encephalitis (*1*). In addition, enteroviruses are well documented as a cause of acute myocarditis, with viruses in the species *Human enterovirus B*, particularly CBVs, being the most common etiologic agents (*2*).

The common chimpanzee (*Pan troglodytes*) and bonobo (*P. paniscus*) are the closest living relatives to humans (*3*). Research has shown that wild chimpanzees from Cameroon excrete different types of enteroviruses in their feces and that some of the enteroviruses are closely related to those known to infect humans (*4*). It has also been shown that fatal myocarditis caused by CBV can occur in other primates, including the orangutan and the snub-nosed monkey (*5**,**6*). We report an incident of likely human-to-chimpanzee enterovirus transmission, which raises the question of whether enteroviruses are regularly transmitted between humans, chimpanzees, and other primates.

## The Case

Chimpanzees at the Copenhagen Zoo (Copenhagen, Denmark) zoo are housed together and allowed contact with each other at all times. In October 2010, respiratory symptoms (coughing, sneezing, and serous nasal discharge) developed in all chimpanzees at the zoo; however, a 7-year-old female chimpanzee was particularly affected. She was a West African chimpanzee (*P. troglodytes verus*) that had been born at the zoo; typical lifespan for such chimpanzees is 50-60 years. Following 36 hours with respiratory symptoms, lethargy, and decreased appetite, the chimpanzee died on October 25.

**Figure 1:**
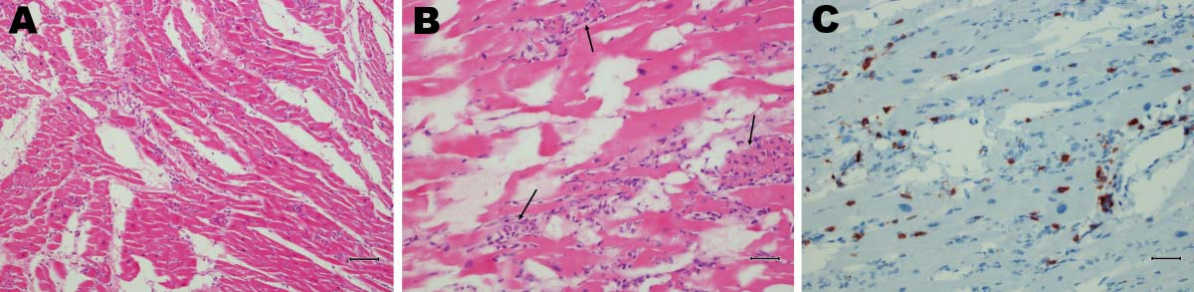
Postmortem tissue sections from chimpanzee with coxsackie virus B infection, Denmark. A) Myocardial section showing artifacts of freezing and a diffuse lymphocytic infiltration. Scale bar = 80 µm. B) Myocytic degeneration (arrows) is evident. Scale bar = 40 µm. C) CD3 marker reaction showing T lymphocytes. Scale bar = 40 µm.

Postmortem examination revealed marked hepatic congestion and mild congestion of the small intestine. The heart was flaccid, and pale streaks were observed in the myocardium. On histographic examination, the lungs showed mild mononuclear interstitial infiltration, and there was marked infiltration in the myocardium consisting mainly of CD3 positive T lymphocytes mixed with a few granulocytes. In addition, multifocal severe myonecrosis was observed; cardiac myocytes were completely engulfed by inflammatory cells (Figure 1).

One-step real-time PCR was performed on RNA extracted from blood, myocardial tissue, and feces to assay for the presence of enterovirus sequences. All results were strongly positive; cycle threshold values of samples were unusually low, in comparison with general values observed for enterovirus-infected samples from humans (*7**,**8*). On the basis of histopathologic and microbiologic findings, we concluded that the chimpanzee died from generalized infection by an enterovirus, which caused an overwhelming inflammation of the heart muscle.

We further characterized the virus by using RNA extracted from the heart. Virus was reverse-transcribed by using SuperScript III First-Strand Synthesis System for RT-PCR (Invitrogen, Carlsbad, CA), and then subjected to PCR amplification by using an enterovirus-specific, long-range PCR approach. A near complete viral genome (≈7,200 nucleotides; 96%) was amplified with primer sequences 5′-GGTGCGAAGAGTCTATTGAGC-3′ and 5′-CACCGAAYGCGGAKAATTTACCCC-3′. PCR amplification was performed by using the Platinum *Taq* DNA Polymerase High Fidelity Kit (Invitrogen). Reactions were performed in 25μL volumes comprised of 1× HiFi buffer, 2 mmol/L MgSO_4_, 0.2 mmol/L dNTPs, 0.4 μmol/L each primer, 1 μL template cDNA, and 1 U enzyme. Cycling conditions were 94°C for 2 min; 35 cycles of 94°C for 30 s, 55°C for 30 s, and 68°C for 7 min 45 s; followed by a final extension at 72°C for 7 min.

**Figure 2:**
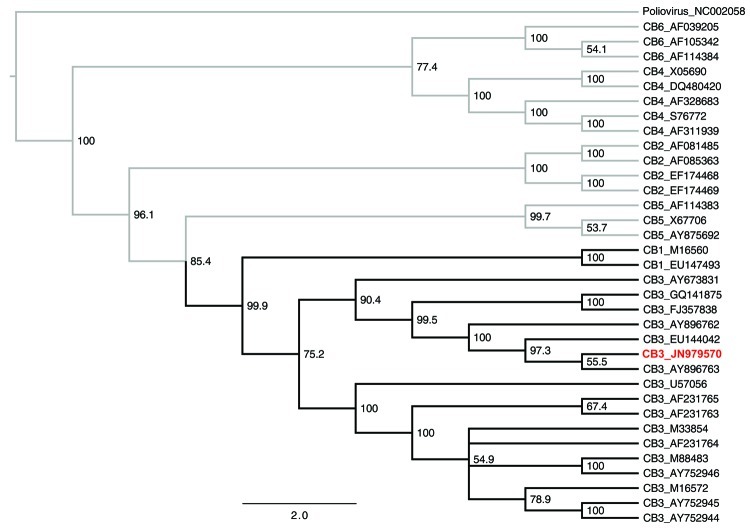
Phylogenetic tree of coxsackie B viruses inferred by using neighbor-joining analysis. The tree was generated by using the Tamura-Nei distance model and 1,000 bootstrap replicates. Scale bar represents estimated phylogenetic divergence. Specific coxsackie B virus serotypes (CB1–6) and corresponding GenBank accession number are shown on the right. Poliovirus was included as an outgroup. Coxsackie virus B clade shown in **boldface**; the reported coxsackie B virus sequence is listed in red. CB, coxsackie B virus.

After PCR amplification, the amplicons were fragmented, converted into a sequencing library, and sequenced by using the Genome Sequencer FLX System (Roche, Copenhagen, Denmark). A local database containing all virus sequences, except those for HIV, retrieved from the Viral Genomes database in GenBank (downloaded June 7, 2011; www.ncbi.nlm.nih.gov/genomes/GenomesHome.cgi?taxid=10239), was constructed, against which all sequence reads were compared by using BLASTn (http://blast.ncbi.nlm.nih.gov/Blast.cgi). This comparison revealed 89,121 sequence reads with similarity to CB3 virus sequences (expectation value <10^−6^). Using a complete genome of a CB3 virus (strain PD, GenBank accession no. AF231765) as reference, we mapped all sequence reads with similarity to enteroviruses by using SMALT software (www.sanger.ac.uk/resources/software/smalt). A consensus sequence constituting the reported CB3 virus was constructed from the mapped reads (GenBank accession no. JN979570). To assess the likelihood of the reported CB3 virus being of human origin, we performed phylogenetic analysis by using a neighbor-joining method. The phylogeny was generated by using published full-genome CBV sequences; all CBV serotypes and the chimpanzee CB3 virus were represented (Figure 2). The phylogeny shows a topology in which the new CB3 virus is clustered within a clade containing human CB3 viruses. Thus, it is most likely that the CB3 virus that infected the female chimpanzee was of human origin rather than a novel type.

In addition, a similarity plot with the chimpanzee CB3 virus protein sequence and the CB3 reference strain protein sequence (GenBank accession no. AAA74400) was generated (data not shown). Overall, the plot showed >95% similarity between the 2 sequences; however, 83% similarity was shown in the 2A region of the sequences. Such a change in the 2A protein could theoretically contribute to altered pathogenicity of the CB3 virus, although whether this is the case would require additional analyses.

## Conclusions

A CB3 virus was detected at extremely high concentration in the heart muscle of a chimpanzee that died from myocarditis. Given the close similarity of the near-complete viral genome sequence to that of human CB3 viruses, the source of the virus was likely a human. Infection of the chimpanzee colony most likely occurred through close contact with an animal keeper or other zoo employee. Alternate explanations, such as transmission by contact between the chimpanzee colony and other zoo animals or zoo visitors, are unlikely because the chimpanzees had no contact with other animals, and they are separated from the public by a 4-m-high glass wall.

Within the *Enterovirus* genus, some enteroviruses that infect humans also infect animals (e.g., polioviruses can infect nonhuman primates and humans), and some primates can be infected with several other enterovirus types. CB5 virus was isolated from an infant chimpanzee with a fatal illness (*12*), and mice can be infected by several coxsackie viruses. Moreover, swine vesicular disease virus, which infects swine, is serotypically identical and antigenically closely related to human coxsackie B5 virus (*9**–**11*).

This report shows that transmission of viruses from humans to chimpanzees is possible and can be fatal. Whether enteroviruses are regularly transmitted between humans, chimpanzees, and other primates, and whether some primates serve as reservoirs for mutation of enteroviruses that can then infect other primates remains to be investigated.
